# Distribution, typology and specificity of pediatric polytrauma centers in Italy

**DOI:** 10.3389/fsurg.2026.1810009

**Published:** 2026-06-17

**Authors:** Simone Frediani, Angelo Zarfati, Pietro Betalli, Marco Gambino, Pietro Impellizzeri, Francesco Morini, Sezione Chirurgia Pediatrica d’Urgenza SICP Study Group, Ivan Pietro Aloi

**Affiliations:** 1 Pediatric General and Urgency Surgery Unit—Bambino Gesù Children’s Hospital IRCCS, Rome, Italy; 2 Pediatric Surgery, Fondazione IRCCS Ca’ Granda Ospedale Maggiore Policlinico, Milan, Italy; 3 Pediatric Surgery Unit, “Annunziata” Hospital, Cosenza, Italy; 4 Department of Human Pathology in Adult and Developmental Age “Gaetano Barresi”—Unit of Paediatric Surgery, University of Messina, Messina, Italy; 5 Pediatric Surgery Unit, Sapienza University of Rome, Azienda Ospedaliero-Universitaria Policlinico Umberto I, Rome, Italy

**Keywords:** children, hepatic trauma, polytrauma, renal trauma, splenic trauma, thoracic trauma

## Abstract

**Introduction:**

Polytrauma is defined by the presence of multiple traumatic injuries and poses a significant global health concern due to its associated morbidity. Clear guidelines or recommendations for the medical or surgical management of pediatric polytrauma patients are currently lacking.

**Aims:**

National Survey of the Management of Paediatric Polytrauma was conducted to clarify and delineate the current national standards in the care paediatric polytrauma patients.

**Material and methods:**

We conducted a survey among senior physicians from the Italian Society of Pediatric Surgery and the Italian Society of Pediatric and Neonatal Anesthesia and Intensive Care. The survey focused on the management of pediatric polytrauma and compared practices between pediatric and hybrid hospitals, as well as between high- and low-volume centers.

**Results:**

A national survey of 52 institutions revealed that 33% were paediatric hospitals and 67% were hybrid institutions treating adult patients. We found that 89% of the institutions had a dedicated paediatric emergency department, and 44% had a senior paediatric surgeon on call. Most hospitals had a dedicated paediatric intensive care unit, 72% had a multidisciplinary trauma team, and 94% had an internal protocol for paediatric polytrauma management. We found no significant differences in the number of second-level paediatric emergency departments, availability of paediatric surgeons, multidisciplinary trauma teams, or imaging modalities. High-volume centres had a higher annual incidence of polytrauma than low-volume centres.

**Conclusion:**

The study provides important insights into the current state of paediatric polytrauma management in the Italian healthcare system. The study emphasised the crucial need for standardisation, specialised training, and the development of evidence-based guidelines. The proposed national trauma registry will be instrumental in facilitating this process and improving the quality of care for paediatric polytrauma patients in Italy. Further research examining the effect of volume, training, and guideline implementation on patient outcomes will significantly enhance this field.

## Introduction

Polytrauma is the simultaneous occurrence of multiple traumatic injuries (1, 2). Despite its widespread use, the definition of polytrauma requires widespread acceptance and validation, particularly among adults (1, 3). Nevertheless, the morbidity and mortality caused by polytrauma remain considerable health concerns worldwide (1, 2, 4, 5). Furthermore, the permanent impact on physical and mental health subsequent to an injury and the general quality of life requires consideration (4, 6).

There are no established guidelines or recommendations for the medical or surgical management of patients with paediatric polytrauma (2). Although recommendations have been developed for isolated trauma (7–10), there are no age-specific recommendations or guidelines for polytrauma management (2), resulting in discrepancies in the global management of polytrauma in paediatric patients with polytrauma. Patients can be treated at various facilities with varying resources.

According to the Italian National Institute of Statistics (ISTAT), the resident population of Italy was approximately 58.9 million inhabitants in 2025 (11). A multicentric consortium of expert institutions revealed the current state of trauma therapy for adults in Italy (12). This study included a comprehensive analysis of networks used to manage polytrauma in adults. However, no specific national study has been conducted on paediatric polytrauma (12). Consequently, there is no information regarding the national state of therapy for paediatric patients with polytrauma. Available European census data indicate a paediatric surgery canter density of approximately one centre per ∼1.1 million inhabitants, with about 3.9 paediatric surgeons per 100,000 children across European systems, including Italy (13).

Therefore, the present study aimed to conduct a survey on paediatric polytrauma management in Italy by the Italian Society of Paediatric Surgery (SICP) and Society of Paediatric and Neonatal Anesthesia and Intensive Care (SARNePI) to clarify and delineate the current national standards in paediatric polytrauma care.

## Methods

An online “home made” questionnaire was developed and distributed to senior medical practitioners in the SICP and SARNePI. The full questionnaire is provided as (Table 1A), including surgeons, intensive care physicians, and anaesthesiologists. We targeted Italian facilities that admitted paediatric patients with polytrauma. The study was approved by the Directorates of the Societies. The survey focuses on medical and surgical units that admit paediatric patients with polytrauma in Italy. For the purposes of this study, a paediatric patient was defined as an individual aged 0–18 years, consistent with commonly adopted international paediatric care definitions.

**Table 1A:** National survey.

Item	Question	Answer
1	What level of care does your hospital provide in trauma management?	a) DEA I level (HUB)
		b) DEA II level (HUB)
		c) Spoke center
2	Is your hospital pediatric-specific center or a ordinary hospital (treats adults as well)?"	a) Pediatric center
		b) Multidisciplinary
3	Does your hospital have a pediatric surgeon present on-site 100% of the time?	a) Yes
		b) No
4	Does your hospital have a pediatric intensive care unit (pICU)?	a) Yes
		b) No
5	Does your hospital have a pediatric anesthesiologist on-site 100% of the time?	a) Yes
		b) No
6	How many cases of pediatric polytrauma (<18 years old) does your hospital receive in a year?	
7	How many traumatic spleen injury cases does your hospital receive in a year (<18 years old)?	
8	How do you treat traumatic splenic injuries?	a) Conservative
		b) Surgical
		c) Endovascular embolization
9	What factors do you consider when selecting surgical treatment for the splenic trauma patient [multiple answer]?	a) AIS score
		b) Haemodynamic instability
		c) Lesion unamenable to embolization
		d) Haemoperitoneum
10	How many traumatic renal injury cases does your hospital receive in a year (<18 years old)?	
11	How do you treat traumatic renal injuries?	a) Conservative
		b) Surgical
		c) Endovascular embolization
12	What factors do you consider when selecting surgical treatment for the renal trauma patient [multiple answer]?	a) AIS score
		b) Haemodinamic instability
		c) Haematuria
		d) Urine extravasation (severity)
		e) Urine extravasation (duration)
13	How many traumatic liver injury cases does your hospital receive in a year (<18 years old)?	
14	How do you treat traumatic liver injuries?	a) Conservative
		b) Surgical
		c) Endovascular embolization
15	What factors do you consider when selecting surgical treatment for the renal trauma patient [multiple answer]?	a) AIS score
		b) Haemodynamic instability
		c) Lesion unamenable to embolization
		d) Haemoperitoneum
		e) Biliary tract injury (severity)
		f) Biliary tract injury (duration)
16	How many traumatic lung injury cases does your hospital receive in a year (<18 years old)?	
17	How do you treat traumatic lung injuries?	a) Conservative
		b) Surgical
		c) Endovascular embolization
18	What factors do you consider when selecting surgical treatment for the lung trauma patient [multiple answer]?	a) AIS score
		b) Haemodynamic instability
		c) Lesion unamenable to embolization
		d) Hemothorax
19	What are the most common reasons that prompt you to transfer a polytraumatized pediatric patient?	a) Necessity og pICU admission
		b) Unavailability of equipment suitable for pediatric patient treatment
		c) Unavailability of competent specialists
		d) Complex injuries of specific organs (es. Liver/pancreas)
20	To which type of center do you refer the patient (most frequent)?	a) Same region
		b) Different region
21	What is the first imaging exam performed on a polytraumatized pediatric patient at your center?	a) eFAST ultrasound
		b) Complete abdominal ultrasound and/or thoracic ultrasound
		c) CT scan
		d) Xray
22	Does your hospital have an internal protocol for the management of polytraumatized pediatric patients?	a) Yes
		b) No
23	Does your hospital have a designated trauma team (anesthesiologist, pediatric surgeon, neurosurgeon, orthopedic surgeon)?	a) Yes
		b) No

The study was conducted using Google Forms. The poll was conducted over 4 months, from 1 April 2023 to 30 July 2023.

The survey focused on the daily patterns of diagnosis, care, and outcomes of paediatric polytrauma. We performed a comparative analysis between paediatric and hybrid hospitals, defined as facilities that treat adult and paediatric patients, and between high- and low-volume centres to assess national variability in paediatric polytrauma management. In the absence of a validated definition (2), as for other pathologies (14, 15), we developed a definition of centres based on volume activities. We arbitrarily defined low-volume centres as those handling ≤24 polytrauma cases annually (i.e., ≤2 cases per month) and high-volume centres as those managing >24 polytrauma cases annually (i.e., >2 cases per month).

In the context of this survey, a Senior Paediatric Surgeon was defined as a board-certified paediatric surgeon with independent clinical responsibility and active involvement in trauma management within the participating institution. For the purposes of this study, paediatric polytrauma was defined as the presence of injuries involving two or more anatomical regions resulting from a single traumatic event. Severe injury was defined as a life-threatening condition requiring immediate or time-sensitive intervention, including physiological instability, the need for emergency surgical or intensive care management, or an Injury Severity Score (ISS) ≥ 16, consistent with commonly adopted trauma system definitions.

Categorical variables were presented as absolute and relative frequencies. Continuous variables were presented as medians and ranges.

### Statistical analysis

Categorical variables were compared using Fisher's exact test or the Chi-square test, as applicable, considering the sample size. We used the Mann–Whitney U test, a non-parametric method, to compare continuous variables. All *P*-values were bilateral. Statistical significance was set at *p* < 0.05.

## Results

Fifty-two institutions in the SICP and SARNePI networks were invited to participate in the national survey, and 36 (69.2%) took up the invitation. Of these, 12 establishments (33%) were paediatric hospitals, and 24 (67%) were hybrid entities that provided care to adult patients.

Thirty-two institutions (89%) had specialised paediatric emergency departments, and 25 and 7 hospitals had second- and first-level paediatric emergency departments, respectively. Conversely, four institutions (11%) had no dedicated paediatric emergency departments. All centres without paediatric emergency departments were hybrid institutions. Sixteen (44%) centres had a senior paediatric surgeon available 24 h. Conversely, 20 hospitals (56%) had senior paediatric surgeons available on call. Additionally, 24 institutions (66%) had specialised paediatric care units.

Twenty-seven (79%) centres had a senior paediatric anaesthesiologist available for 24 h at the hospital, whereas the remaining centres had one on-call. Twenty-six (72%) institutions (72%) had a dedicated multidisciplinary trauma team. Thirty-four hospitals (94%) implemented an internal policy for multidisciplinary paediatric polytrauma management. Twenty-eight (77%), 5 (13%), and 5 (13%) institutions utilised ultrasonography, radiography, and computed tomography (CT) as their principal imaging modality, respectively.

The facilities treated a median of 20 patients with paediatric polytrauma annually, with an annual range of 5–200 cases. We classified 20 (56%) and 16 (44%) institutions as low- and high-volume centres, respectively. An annual median of six splenic traumatic injuries was managed, with a range of 2–50 patients annually. An annual median of 5 renal traumatic injuries was managed (an annual range of 1–30 patients). An annual median of 5 hepatic traumatic injuries were treated annually (a range of 1–43 individuals annually). Additionally, a median of four thoracic trauma annual cases was treated (a range of 0–60 patients annually).

Table 1B delineates a comparison between paediatric and hybrid facilities. No significant differences in the total dedicated paediatric emergency departments between the two categories were observed. Paediatric institutions possessed a significantly higher number of second-level paediatric emergency departments [11 (92%)] than hybrid facilities [14 (58%): *p* = 0.050]. The two groups exhibited similarities in the primary paediatric emergency departments. No significant difference in the 24 h availability of the paediatric surgeon were present among the groups. Paediatric institutions had a markedly more significant proportion of paediatric anaesthesiologists [12 (100%)] than hybrid facilities [16 (66%), *p* = 0.033]. Additionally, we observed a markedly higher prevalence of specialised paediatric intensive care units in the paediatric institutions (11 [92%] than that in the hybrid facilities [13 (54%), *p* = 0.030]. We observed no significant differences in the presence of a multidisciplinary trauma team and the availability of a management protocol for paediatric polytrauma between the different facilities. The different facilities had no considerable differences in initial imaging techniques used, such as ultrasonography, CT, and radiography. We observed no significant changes in the distribution of paediatric and hybrid institutions between high- and low-volume categories. Furthermore, we found no significant differences between the groups regarding the median annual activity in the treatment of patients with polytrauma. Moreover, the groups exhibited similarities in the number of splenic, renal, hepatic, and thoracic injuries treated.

**Table 1B:** A comparative analysis of pediatric and hybrid institutions.

Item		Total (*n* = 36)	Pediatric (*n* = 12)	Hybrid (*n* = 24)	*p* value
Pediatric emergency department	*First level*	7 (19%)	1 (8%)	6 (25%)	0.383
	*Second level*	25 (69%)	11 (92%)	14 (58%)	0.050
	*No*	4 (11%)	0 (0%)	4 (16%)	0.274
Pediatric surgeon	*In hospital 24/7*	16 (45%)	8 (66%)	8 (34%)	0.081
	*On call*	20 (55%)	4 (34%)	16 (66%)	0.081
Pediatric anesthesiologist	*In hospital 24/7*	27 (75%)	12 (100%)	15 (66%)	0.033
	*On call*	9 (25%)	0 (0%)	9 (34%)	0.033
Pediatric intensive care unit		24 (66%)	11 (92%)	13 (54%)	0.030
Multidisciplinary trauma team		26 (72%)	7 (58%)	19 (79%)	0.247
Protocol for polytrauma management		34 (94%)	11 (92%)	23 (95%)	1.000
Volume of activity	*High-volume*	16 (45%)	6 (50%)	10 (41%)	0.729
	*Low-volume*	20 (55%)	6 (50%)	14 (58%)	0.729
Median polytrauma/year (range)		20 (5–200)	20 (10–100)	20 (5–200)	0.818
Median splenic trauma/year (range)		6 (2–50)	10 (4–20)	5 (2–50)	0.067
Median renal trauma/year (range)		5 (1–30)	5.5 (1–15)	3.5 (1–30)	0.067
Median hepatic trauma/year (range)		5 (1–43)	6 (3–10)	4.5 (1–43)	0.226
Median thoracic trauma/year (range)		4 (0–60)	5 (2–10)	3 (0–60)	0.118
First line imaging	*Ultrasound*	28 (77%)	10 (83%)	18 (78%)	0.691
	*CT*	13 (36%)	4 (34%)	9 (37%)	1.000
	*x-rays*	5 (13%)	2 (16%)	3 (12%)	1.000

Table 1C presents a comparison between the high- and low-volume centres. The groups exhibited similarities in the presence of a paediatric emergency department and its level of activity. Moreover, we observed a comparable number of paediatric surgeons and anaesthesiologists in the hospitals. The hospitals exhibited similarities in the presence of a multidisciplinary trauma team and a paediatric intensive care unit. Moreover, we observed no significant differences in the existence of paediatric polytrauma care protocols. We observed no significant variations across the groups regarding the initial imaging in the protocol. High-volume centres exhibited a markedly greater median incidence of polytrauma each year than low-volume centres [10 [range: 5–20] vs. 50 [range: 25–200]; *p* < 0.0001]. Additionally, we observed a significantly higher median annual incidence of splenic [4.5 [range: 2–10] vs. 10 [range: 4–50]: *p* = 0.0010], renal [2.5 [range: 1–10] vs. 5 [range: 1–30]; *p* = 0.0251], hepatic [3.5 [range: 1–10] vs. 6.5 [range: 1–43]; *p* = 0.0226], and thoracic traumas [2 [range: 0–10] vs. 6 [range: 2–60]; *p* = 0.0009] in the high-volume centres compared to that in the low-volume centres.

**Table 1C:** Comparison between high-volume and low-volume center institutions.

Item		Total (*n* = 36)	Low-volume (*n* = 20)	High-volume (*n* = 16)	*p* value
Pediatric emergency department	*First level*	7 (19%)	5 (25%)	2 (12%)	0.426
	*Second level*	25 (69%)	11 (55%)	14 (87%)	0.067
	*No*	4 (11%)	4 (20%)	0 (0%)	0.113
Pediatric surgeon	*In hospital*	16 (45%)	10 (50%)	6 (37%)	0.515
	*On call*	20 (55%)	10 (50%)	10 (63%)	0.515
Pediatric anesthesiologist	*In hospital*	27 (75%)	13 (65%)	14 (87%)	0.244
	*On call*	9 (25%)	7 (35%)	2 (12%)	0.244
Pediatric intensive care unit		24 (66%)	12 (60%)	12 (75%)	0.481
Multidisciplinary trauma team		26 (72%)	12 (60%)	14 (87%)	0.132
Protocol for polytrauma management		34 (94%)	18 (90%)	16 (100%)	0.492
Type of hospital	*Pediatric*	12 (33%)	6 (30%)	6 (37%)	0.729
	*Hybrid*	24 (66%)	14 (70%)	10 (63%)	0.729
Median polytrauma/year (range)		20 (5–200)	10 (5–20)	50 (25–200)	<0.0001
Median splenic trauma/year (range)		6 (2–50)	4.5 (2–10)	10 (4–50)	0.0010
Median renal trauma/year (range)		5 (1–30)	2.5 (1–10)	5 (1–30)	0.0251
Median hepatic trauma/year (range)		5 (1–43)	3.5 (1–10)	6.5 (1–43)	0.0226
Median thoracic trauma/year (range)		4 (0–60)	2 (0–10)	6 (2–60)	0.0009
First line imaging	*Ultrasound*	28 (77%)	15 (75%)	13 (81%)	0.708
	*CT*	13 (36%)	7 (35%)	6 (37%)	1.000
	*x-rays*	5 (13%)	2 (10%)	3 (18%)	0.637

## Discussion

This study presents a valuable snapshot of the current state of paediatric polytrauma management in the healthcare system in Italy. The organization of paediatric trauma care in Italy differs from that of several other European countries, where hybrid trauma systems integrating adult and paediatric care are more commonly implemented. In Northern European healthcare models, paediatric trauma patients are frequently managed within integrated trauma networks that include both dedicated paediatric expertise and shared adult trauma resources. Conversely, the Italian system traditionally maintains a clearer separation between paediatric-dedicated hospitals and general trauma centres. This structural distinction may contribute to the variability observed in trauma pathways and resource availability across institutions and highlights the ongoing need for harmonization of paediatric trauma care at a national and European level. The national survey provided insights into the variations in practices across different facilities and highlighted critical areas for improvement. This study appropriately differentiated between paediatric-specific hospitals and hybrid facilities, revealing significant disparities in resource availability and expertise.

One of the most relevant findings emerging from this survey is the absence of a national paediatric trauma registry in Italy. Despite being a high-income country with an established healthcare system, the lack of standardized nationwide data collection limits the ability to accurately monitor epidemiology, evaluate outcomes, and implement evidence-based improvements in paediatric trauma care. The establishment of a national paediatric trauma registry would represent a crucial step toward harmonizing clinical pathways, improving quality assessment, and supporting multicentre research initiatives. Such a registry could also facilitate benchmarking with other European trauma systems and contribute to the development of standardized paediatric trauma networks.

### Paediatric vs. hybrid institutions

A significant difference was noted between paediatric-only and hybrid facilities; paediatric-only hospitals demonstrated a significantly higher prevalence of dedicated paediatric emergency departments, 24 h a day 7 days a week access to paediatric surgeons, anaesthesiologists, and dedicated paediatric intensive care units, consistent with the expected concentration of experts in settings dedicated solely to paediatric care, underscoring the critical role of specialised centres for paediatric polytrauma management. The lack of resources in hybrid hospitals may necessitate a rapid transfer to better-equipped facilities, potentially delaying critical interventions and negatively affecting patient outcomes (14, 15), highlighting the critical need for a structured regionalised system for paediatric trauma care, and facilitating the efficient transfer of patients to specialised units when necessary (16).

An important consideration in paediatric trauma organization relates to age-dependent clinical needs. Although older adolescents may often be effectively managed within adult trauma systems due to comparable physiological characteristics, infants and younger children present unique anatomical, physiological, and anaesthetic challenges that require specialized paediatric expertise. The management of these patients frequently necessitates the availability of paediatric surgeons, paediatric anaesthesiologists, and paediatric intensive care units. Consequently, trauma system planning should account not only for injury severity but also for patient age, supporting the development of paediatric-specific pathways particularly for younger age groups.

### High-volume vs. low-volume centres

Analysis of high- and low-volume centres revealed substantial differences in the annual caseload and severity of injuries managed. High-volume centres have higher annual polytrauma cases than low-volume centres, including a significantly higher number of severe injuries (splenic, renal, hepatic, and thoracic traumas), suggesting that high-case-load hospitals have a greater potential to develop enhanced expertise, to plan adequate training for young specialists and potentially achieve better patient outcomes, owing to increased experience and specialised procedures (17). However, this study did not quantify the differences in outcomes, which is an important area for future research. Understanding whether a higher volume results in improved outcomes or reflects the referral patterns is crucial. Prospective studies examining outcomes across volume groups are necessary in the future.

### Standardisation of practices

This study underscores the need for the national standardisation of several aspects of paediatric polytrauma management. Although a high percentage of institutions reported having internal protocols and multidisciplinary trauma teams, variations existed. The standardisation of imaging protocols (e.g., prioritising ultrasound) and surgical treatment strategies across institutions is essential for ensuring consistent diagnostic accuracy and appropriate interventions. A national trauma registry is a vital step towards such standardisation, enabling systematic collection and analysis of data to identify best practices and areas of improvement (18).

The present study focuses on organizational aspects of paediatric trauma care and does not include analysis of patient-level clinical outcomes. Consequently, it was not possible to evaluate potential differences in outcomes between high-volume and low-volume centres. This represents an important limitation, as previous studies in trauma systems have suggested that institutional experience and case volume may influence patient outcomes. Future investigations integrating organizational surveys with standardized national trauma registry data will be essential to determine whether differences in resource availability and centre volume translate into measurable improvements in paediatric trauma outcomes.

### Challenges and future directions

This study highlighted several key challenges. The lack of established national guidelines or recommendations specific to paediatric polytrauma has created inconsistencies in healthcare. The development of clear, evidence-based guidelines encompassing all aspects of paediatric polytrauma management, from initial assessment to rehabilitation, should be prioritised (19).

Although the present survey focused primarily on abdominal solid organ injuries, paediatric polytrauma management inherently requires a multidisciplinary approach involving multiple organ systems. Severe paediatric trauma frequently includes thoracic, neurological, and orthopaedic injuries, each requiring specific expertise and coordinated management. The availability of trained specialists, including neurosurgeons, orthopaedic surgeons, paediatric intensivists, and dedicated trauma teams, represents a critical component of high-quality paediatric trauma care. Therefore, evaluation of trauma centre preparedness should extend beyond single-organ management and consider the broader institutional capacity to address multisystem injuries in children.

Furthermore, specialised training programs for healthcare providers are essential for improving their competency in handling paediatric trauma cases. The survey findings revealed that the availability of specialised personnel, such as paediatric anaesthesiologists, varied across facilities. Ensuring adequate training and access to continuing medical education for paediatric surgeons, anaesthesiologists, and other relevant healthcare professionals is crucial for building a more robust system (20).

### Integration with existing trauma systems

The findings of this study should be applied to existing trauma systems and initiatives in Italy, providing a more holistic view of trauma care in Italy and helping optimise resource allocation and training programs across the trauma care spectrum (21).

Understanding how data align with the adult trauma care pathways is essential. Furthermore, there is a need to elucidate whether the challenges faced by adults with polytrauma are the same as those of the paediatric population.

## Conclusion

This study provides important insights into the current state of paediatric polytrauma management in the Italian healthcare system. It highlights significant disparities between paediatric-only and hybrid facilities and between high- and low-volume centres, emphasising the crucial need for standardisation, specialised training, and the development of evidence-based guidelines. The proposed national trauma registry will be instrumental in facilitating this process and improving the quality of care for paediatric polytrauma patients in Italy. Further research examining the effect of volume, training, and guideline implementation on patient outcomes will significantly enhance this field.

## Data Availability

The original contributions presented in the study are included in the article/Supplementary Material, further inquiries can be directed to the corresponding author.

## Group members of Sezione Chirurgia Pediatrica d'Urgenza SICP Study Group

Department of Pediatric Surgery, ASST Spedali Civili, Brescia, Italy: Alberti Daniele, Romeo Carmelo, Boroni Giovanni. Department of Human Pathology of Adult and Childhood “Gaetano Barresi”, University of Messina, Messina, Italy: Antonuccio Pietro, Salvatore Arena. Department of Pediatric Surgery and Urology Unit, San Camillo Forlanini Hospital, Rome, Italy: Briganti Vito, Oriolo Lucia. Department of Pediatric Surgery, Ospedale Garibaldi, Catania, Italy: Cacciaguerra Sebastiano, Iacona Roberta Valentina. Pediatric Surgery Unit, San Carlo Regional Hospital, Potenza, Italy: Caiazzo Paolo, Del Prete Immacolata. Pediatric Surgery Department, Policlinico di Modena, Modena, Italy: Ceccarelli Pier Luca, Paolo Repetto. Department of Pediatric Surgery, ASST Papa Giovanni XXIII Hospital, Bergamo, Italy: Cheli Maurizio, Giovanni Parente. Division of Pediatric Surgery, San Bortolo Hospital, Vicenza, Italy: Chiarenza Fabio, Cosimo Bleve. Bambino Gesù Children's Hospital IRCCS, Rome, Italy: Bagolan Pietro; Alessandro Inserra, Conforti Andrea, Reali Simone. Department of Paediatric Surgery, Meyer Children's Hospital IRCCS, Florence, Italy: Morabito Antonino, Riccardo Coletta. Pediatric Surgery Unit, Department of Health Promotion, Mother and Child Care, Internal Medicine and Medical Specialties “G D'Alessandro”, University of Palermo, Palermo, Italy: Di Pace Maria Rita, Sergio Maria. Department of Pediatrics, Santa Maria delle Croci Hospital, 48121 Ravenna, Italy: Domenichelli Vincenzo. Veronica Carlini. Department of Translational Medical Sciences, Federico II University, Naples, Italy: Esposito Ciro. Unit of Pediatric Surgery, Woman and Child Department, Filippo Del Ponte Hospital - ASST Sette Laghi, Varese, Italy: Gentilino Valerio, Farris Giorgio. Pediatric Surgery, Fondazione IRCCS Ca’ Granda Ospedale Maggiore Policlinico, Milan, Italy: Leva Ernesto, Cardarelli Mery. Pediatric Surgery Unit, Sapienza University of Rome, Azienda Ospedaliero-Universitaria Policlinico Umberto I, Rome, Italy: Cozzi DA., Ceccanti Silvia. Department of Pediatric Surgery, Fondazione IRCCS Ca’ Granda Ospedale Maggiore Policlinico, Milan, Italy: Macchini Francesco, Zanini Andrea. Paediatric Surgery Unit, Cà Foncello Hospital, Treviso, Italy: Midrio Paola, Gobbi Paola. Pediatric Surgery, IRCCS, Policlinico Universitario AGemelli, Rome, Italy: Nanni Lorenzo, Beati Federico. Pediatric Surgery Unit, Fondazione IRCCS Policlinico San Matteo, University of Pavia, Pavia, Italy: Riccipetitoni Giovanna. Pediatric Surgery, Azienda Ospedaliero Universitaria “Maggiore Della Carità”, Novara, Italy: Ubertazzi Michele, Gabriele Valeria. Pediatric Surgical Unit, SS Trinità Hospital, Cagliari, Italy: Garau Roberto, Atzori Pietro. Pediatric Surgery Unit, Department of Medical and Surgical Sciences, University of Foggia, Foggia, Italy: Canale Francesco, Raffaella Cocomazzi. Pediatric Surgery Unit, “S Antonio Abate” Hospital, Trapani: Formica Paolo, Milana Erminio. Pediatric Surgery Department, Vittore Buzzi Children's Hospital, Milano, Italy: Pellizzo Gloria, Costanzo Sara, Campari Alessandro. Department of Pediatric Surgery, Umberto Bosio Center for Digestive Diseases, The Children Hospital AO SS Antonio E Biagio E Cesare Arrigo, Alessandria, Italy: Incerti Filippo, Pagliara Camilla. Department of Pediatric Surgery and Urology, Institute for Maternal and Child Health-IRCCS “Burlo Garofolo”, Trieste, Italy: Codrich Daniela. Department of Radiology, Sant'Anna Hospital, AOU Città della Salute e della Scienza, Turin, Italy: Stefano Gamba, Hassan Alhellani. Paediatric Surgery Department, AORN Santobono Pausilipon, Napoli, Italy: Grella Maria Giovanna, Coppola Sandra. Pediatric Surgery, Women's and Children's Health Department, University of Padua, Padua, Italy: La Pergola Enrico, Volpe Andrea. Pediatric Surgery, Policlinico Santa Maria alle Scotte, Siena, Italy: Angotti Rossella, Nascimben Francesca.
